# Comparative Metabolomics of Ligulate and Tubular Flowers of Two Cultivars of *Calendula officinalis* L.

**DOI:** 10.3390/metabo14030140

**Published:** 2024-02-26

**Authors:** Vladimir Ossipov, Firdaus Khazieva, Dmitry Baleev, Juha-Pekka Salminen, Nikolay Sidelnikov

**Affiliations:** 1 All-Russian Institute of Medicinal and Aromatic Plants, Grina 7, Moscow 117216, Russia; vilar.6@yandex.ru (F.K.); dbaleev@gmail.com (D.B.); vilarnii@mail.ru (N.S.); 2 Natural Chemistry Research Group, Department of Chemistry, University of Turku, FI-20014 Turku, Finland; j-p.salminen@utu.fi

**Keywords:** ‘Golden Sea’, ‘Paradise Garden’, biologically active metabolites, UPLC-PDA-HRMS, ligulate and tubular flowers, phenolic compounds, triterpenoid glycosides, lipids

## Abstract

*Calendula officinalis* L. is a well-known plant widely used in traditional medicine due to the presence of various biologically active compounds. The main raw material for the production of medicinal preparations is the inflorescence, which consists of ligulate and tubular flowers. However, the characteristics of the metabolome of these flowers are not fully understood. This study identified and compared the levels of major metabolites in the ligulate and tubular flowers of two *C. officinalis* cultivars, ‘Golden Sea’ (GS) and ‘Paradise Garden’ (PG). The metabolome was analysed using ultra-performance liquid chromatography with photodiode array detection and a Q Exactive Orbitrap high-resolution mass spectrometer. It was found that the tubular flowers of both PG and GS cultivars had higher levels of lipids, phenolamides and caffeoylquinic acids and lower levels of triterpenoid glycosides than the ligulate flowers. It was also shown that the inflorescences of the GS, which had a 35% higher proportion of tubular flowers, contained 30% more phenolic compounds and 50% more lipids than the PG. Thus, the results obtained extend our understanding of the features in the metabolomes of ligulate and tubular flowers and suggest that the quality of inflorescences of *C. officinalis* cultivars, as a source of medicinal preparations, is strongly influenced by the proportion of ligulate and tubular flowers.

## 1. Introduction

*Calendula officinalis* L., commonly known as pot marigold, is an annual plant that belongs to the Asteraceae family. It has been used in traditional medicine for centuries [1]. Preparations made from *C. officinalis* have been shown to exhibit a wide range of pharmacological activities [2,3,4]. The therapeutic properties of *C. officinalis* are attributed to the presence of biologically active compounds. Among these, phenolic compounds and triterpenoids are the most abundant [5]. 

*C. officinalis* contains a range of phenolic compounds, such as flavonoids, cinnamic and benzoic acids, coumarins, anthocyanins and their derivatives [5,6]. Research has demonstrated that these phenolics possess antimicrobial, antifungal and antiviral properties [6,7], as well as strong antioxidant activity [4,8,9]. Consuming phenolic compounds from medicinal plants or food can significantly reduce the risk of many diseases associated with oxidative stress, including cancer, diabetes, cardiovascular diseases, rheumatoid arthritis, Alzheimer’s and Parkinson’s diseases [10,11]. 

The triterpenoids of *C. officinalis* can be classified into two groups based on their solubility in water and organic solvents [12]. The first group includes the water-soluble oleanolic acid glycosides, which differ in the number and position of the attached glucose, galactose and glucuronic acids [8,9,13]. For *C. officinalis*, the characteristic triterpenoid glycosides are calendulaglycosides A, B and C, and calendulosides E, F, G, H and E [3]. The second group of triterpenoids is only soluble in organic solvents such as chloroform, dichloromethane and n-hexane. This group includes free triterpenoids and their fatty acid esters [12,13,14]. The triterpenoids of *C. officinalis* exhibit anti-inflammatory, anti-allergic, anti-ulcer, cytotoxic, anti-tumour, anti-mutagenic and anti-diabetic activities [3,15,16]. 

Compared to phenolic compounds and triterpenoids, the lipids in *C. officinalis* inflorescences have been poorly studied, except for carotenoids [1,4]. The composition and relative content of fatty acids are known [17]. The inflorescences have a predominance of saturated acids (77%), mainly palmitic (C16:0, 36%) and myristic (C14:0, 25%). Linolenic acid (C18:3n3, 46%) is the most abundant polyunsaturated acid [18]. The lipids of *C. officinalis* have been extensively studied in the seeds due to their high content, ranging from 5% to 22% [18]. They consist of phospholipids, glycolipids, neutral lipids and fatty acids [2,14]. One of the most important fatty acids found in the seeds is α-calendic acid, which belongs to the class of conjugated octadecatrienoic or linolenic acids [19]. Seed lipids can contain up to 60% of α-calendic acid, according to [20]. However, the content of this acid is much lower in the leaves and inflorescences. It is known that conjugated linolenic acids have a wide range of biological activities. These acids can regulate human lipid metabolism and provide anti-inflammatory and antioxidant properties [21,22,23,24].

The main raw material of *C. officinalis* for the production of medicinal preparations is the inflorescences, which contain the largest amounts of biologically active compounds [25,26]. The inflorescences consist of ligulate and tubular flowers, which differ not only morphologically and in physiological function, but also in the composition and content of metabolites [26,27,28,29]. However, to date, comparative phytochemical studies of ligulate and tubular flowers have been limited to phenolic compounds [26,27,28], essential oils [27,28,29] and triterpenoid esters [30], and the results obtained have been contradictory. For instance, one study on phenolic compounds found the highest levels of flavonoids in ligulate flowers [26], while another study found them in tubular flowers [27]. Uncertain results have also been obtained for the content of essential oils [26,29]. A detailed analysis of phenolic compounds showed that tubular flowers have higher levels of caffeoylquinic acids and anthocyanins, while ligulate flowers have higher levels of flavonoids [28]. The study by Zitterl-Eglseer et al. [30] found that ligulate flowers had a significantly higher amount of triterpenoid esters compared to tubular flowers. 

In addition, the proportion of ligulate and tubular flowers in inflorescences may vary depending on the cultivar, climate, soil and agronomic conditions [26,31]. It is important to note that these variations are influenced by external factors and may not be consistent across different cultivars or growing conditions. For example, in a study of five cultivars of *C. officinalis*, the percentage of ligulate flowers in the inflorescences ranged from 51% to 68%, while the percentage of tubular flowers ranged from 10% to 16% [26]. According to [31], the proportion of ligulate flowers compared to tubular flowers in the *C. officinalis* cultivar ‘Orange’ varied from 13% to 62% in years with different climatic conditions. This variation may strongly influence the phytochemical quality of *C. officinalis* inflorescences as a source of medicinal preparations, if the ligulate and tubular flowers differ significantly in metabolite content. 

Therefore, the main objective of this study was to identify and compare the major metabolites of ligulate and tubular flowers of two cultivars of *C. officinalis*, ‘Golden Sea’ and ‘Paradise Garden’. In addition, the effect of different proportions of ligulate and tubular flowers on the content of these metabolites in the inflorescences of the cultivars was investigated. The metabolome of the flowers was analysed using ultra-performance liquid chromatography with photodiode array detection and a Q Exactive Orbitrap high-resolution mass spectrometer.

## 2. Materials and Methods

### 2.1. Characteristics of Plant Objects

**Figure 1:**
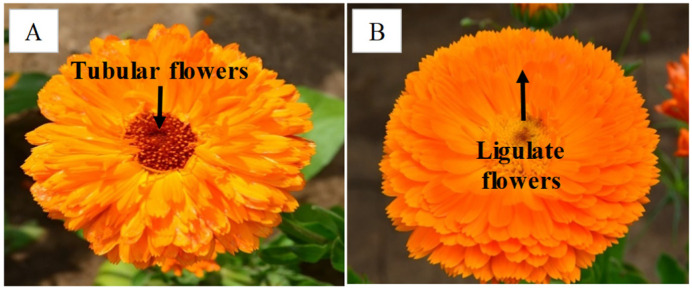
Photo of *Calendula officinalis* inflorescences of cultivars ‘Golden Sea’ (**A**) and ‘Paradise Garden’ (**B**).

The objects of this study were the ligulate and tubular flowers of two cultivars of *Calendula officinalis* L., namely ‘Golden Sea’ (GS) and ‘Paradise Garden’ (PG) (Figure 1). These cultivars were obtained through chemical mutagenesis of the original cultivar ‘Kalta’, which had lost many valuable characteristics over years of cultivation [32]. The Russian cultivar names in Latin transliteration are GS—‘Zolotoe more’ and PG—‘Rajiskij sad’. When comparing the resulting cultivars for morphological traits, it was observed that the GS plants had a higher seed production, a greater number of leaves and a 38% higher proportion of tubular flowers in their inflorescences compared to the PG cultivars (Table S1).

The cultivars of *C. officinalis* were grown in the Botanical Garden of the All-Russian Institute of Medicinal and Aromatic Plants in Moscow, Russia. The seeds were sown in early spring using the wide-row sowing method with a row spacing of 60–70 cm, a seed rate of 8–10 kg/ha and a sowing depth of 2–3 cm. The experimental plots were covered with sodo-podzol medium-podzol dusty loams (80–100 cm thick) underlain by moraine deposits. The soil’s arable layer has a brownish-grey colour and a fine, lumpy or clumpy texture, with a thickness of 22–23 cm. Its granulometric composition is medium-loamy, and it contains more than 40–50% of water-resistant aggregates (>0.5 mm) that are agronomically valuable. The soil’s agrochemical parameters are as follows: humus content—2.1%; mobile phosphorus P_2_O_5_—52 mg/kg; exchangeable potassium K_2_O—87 mg/kg; and pH—5.5 [32]. 

Four samples of inflorescences, each weighing approximately 100 g, were collected from the experimental plots of both cultivars in mid-July during the flowering period. The inflorescences were dried in the dark in a ventilated thermostat at 45 °C and separated into ligulate and tubular flowers. The dry flowers were weighed and homogenised using an MM 200 ball mill (Retsch GmbH & Co. KG) for 2 min at 30 Hz. In total, there were sixteen biological samples of the ligulate and tubular flowers: eight samples of the GS cultivar and eight samples of the PG cultivar.

### 2.2. UPLC-PDA-HRMS Analysis of Metabolites

For quality control (QC) purposes, three technical replicates of each biological flower sample weighing 10 ± 1 mg were extracted with 1 mL of 80% methanol containing internal standards: lidocaine (*m*/*z* 235.1803 [M+H]^+^, detected in positive ion mode; 5 mg/L) and (1*R*)-(-)-10-camphorsulphonic acid (*m*/*z* 231.0686 [M-H]^−^, detected in negative ion mode; 5 mg/L) [33]. The extraction was carried out for 60 min at room temperature with constant stirring (VORTEX Genie 2, Scientific Industries, Bohemia, NY, USA). The resulting extracts were separated by centrifugation (10 min at 20,000× *g*) and filtered through a syringe filter (4 mm, 0.2 μm PTFE, Thermo Fisher Scientific Inc., Waltham, MA, USA). A total of forty-eight flower samples were prepared for UPLC-PDA-HRMS metabolite analysis. In addition to forty-eight samples of flower extracts, the master sample was prepared for QC. This sample was obtained by pooling aliquots (0.1 mL) from all the flower extracts. 

The UPLC-PDA-HRMS system consisted of an ultra-performance liquid chromatograph with a UV-Vis photodiode array detector (PDA, 190–500 nm) (Acquity UPLC^®^ 2.9.0, Waters Corporation, Milford, MA, USA) and a high-resolution Q Exactive Orbitrap mass spectrometer (Thermo Fisher Scientific Inc., Waltham, MA, USA). The mass spectrometer was equipped with a heated electrospray ionisation (HESI) source operating in negative or positive ionisation mode, scanning ions in the *m*/*z* 150–2000 range. The HESI conditions were as follows: the sheath gas flow rate was set at 60, the auxiliary gas flow rate was set at 20, arbitrary units were set by Tune software, the spray voltage was set at 3 kV, the capillary temperature was set at 380 °C and the S-lens RF level was set at 60.0. The settings for full-scan mode were as follows: microscans 1, resolution of 140,000 FWHM and 34,600 FWHM (data-dependent MS/MS), AGC target of 3 × 106 and maximum IT of 200 ms. The instrument was operated using XCalibur 3.0.63 software (Thermo Fisher Scientific Inc., Waltham, MA, USA) [33]. 

The separation of metabolites from *C. officinalis* flowers was performed on an Acquity UPLC^®^ BEH column (2.1 × 100 mm, 1.7 µm, Waters Corporation, Ireland) using two eluents: (A) 0.1% aqueous formic acid solution and (B) acetonitrile containing 0.1% formic acid. Gradient program: 0–0.5 min, 0.1% B in A; 0.5–10.0 min, 0.1–95.0% B in A (linear gradient); 10.0–13.0 min, 95.0% B in A (isocratic mode); 13–15 min, column wash and stabilisation. The eluent flow rate was 0.5 mL/min. The sample-injected volume was 5 µL [33].

During UPLC-PDA-HRMS analysis, the extract samples were randomly distributed. For QC purposes, a blank (water) and a master sample were analysed twice at the beginning, middle and end of the total sample set.

### 2.3. Processing of the MS Data 

XCMS Online software 3.7.1 (xcmsonline.scripps.edu) was used for the initial processing of MS data [34]. Raw MS chromatogram files in negative or positive ionisation mode of the metabolites were converted to NetCDF format, exported to XCMS Online software and analysed individually according to integrated method ‘UPLC/Orbitrap’. The method parameters for file processing included the following: 1. Feature detection. ppm—5; minimum and maximum peak width—5 and 20; prefilter peaks—3; S/N threshold—100; mzdiff—0.01; integration method—1; prefilter intensity—10,000; noise filter—50,000. 2. Retention time correction. Obiwarp method; profStep—1. 3. Alignment. mzwid—0.025; minfrac—0.5; bw—5; max—100; minsamp 1. The program automatically corrected the baseline of the chromatograms, determined the peaks of the metabolites, aligned their positions on the chromatograms of all samples, determined the MS characteristics of the metabolites and performed their preliminary statistical evaluation [34]. 

### 2.4. Bioinfomatic Analysis of the MS Data 

The processed MS data were exported to Excel software and two matrices were generated for negative and positive ions. The matrices contained the intensities of detected *m*/*z* ions in ligulate and tubular flower samples from two *C. officinalis* cultivars. The *m*/*z* ion intensity data were normalised relative to the signal intensity of internal standards for positive and negative ions and sample weight. 

The data were exported to the SIMCA-P+ software package (version 15, Umetrics, Umea, Sweden), mean-centred and unit-variance-scaled for multivariate analysis [33]. Outlier variables were identified using the Hotelling’s T2 ellipse (95% confidence interval) and the distance to the model parameter (DModX). Any detected outliers were carefully examined and, if necessary, removed from the dataset. Means for biological sample replicates were calculated and used to perform Principal Component Analysis (PCA) as an initial overview of sample classification. For the visualisation of the highest and lowest values in the data matrix, a heatmap was also applied [35]. 

In the next step, the raw data were Pareto-scaled and analysed using a supervised classification method—Orthogonal Partial Least Squares to Latent Structures analysis (OPLS). This method focuses on multivariate analyses to identify differences due to the factor under investigation alone. The OPLS method enables the separation of group variability in the plant metabolome into predictive and non-predictive variations within the compared groups. In our experiment, these were differences in the metabolome of two flower types or two cultivars of *C. officinalis* (Figure 1). The significance of the differences between the sample groups was determined using ANOVA of cross-validated predictive residuals (CV-ANOVA) [33]. 

The significance of the differences in the content of individual metabolites was determined using the values of the correlation coefficient with the orthogonal component of the OPLS model, which determines the reliability of the contribution of the metabolite to the discrimination between groups of samples. Correlation values with orthogonal components were obtained from S-plot data of OPLS models. Metabolites with the highest correlation values (*p* > 0.8 or *p* < −0.8) were considered as potential markers determining the difference in the metabolome of the compared groups of *C. officinalis* samples. Student’s paired *t*-test was also used to assess the significance of differences in metabolite levels [33].

### 2.5. Characterisation of Metabolites

To identify the metabolites of *C. officinalis* flowers, mass spectra were analysed and the *m*/*z* values of [M-H]^−^ or [M+H]^+^ ions and their MS/MS fragmentation were determined. From these data, the chemical formulae of the metabolites and their monoisotopic mass (Da) were determined. The original XCalibure programme (version 3.0.63, Thermo Fisher Scientific Inc., Waltham, MA, USA) and the mzMine-3 programme were used [36]. 

The resulting MS and MS/MS data were used for metabolite identification by comparison with data from available MS databases such as Metlin (https://metlin.scripps.edu) [37], Human Metabolome Database (https://hmdb.ca) [38] and Lipid Maps (http://www.lipidmaps.org) [39], as well as with data published in the literature [3,5,28,40,41,42]. The maximum MS error in metabolite identification was within ±1.5 ppm for phenolic compounds and triterpenoids and within ±2.4 ppm for lipids. The metabolite identification results are presented according to the requirements previously developed by ‘The Metabolomics Standard Initiative group’ [43]. In addition to the MS data, the features of the UV spectra were used to characterise the phenolic compounds.

### 2.6. Quantitation of Metabolites

The most intense *m*/*z* ion value of the mass spectrum, mainly [M-H]^−^ or [M+H]^+^, was normalised against an internal standard and sample mass and used to characterise the relative metabolite content in the flower sample. The results are expressed in relative units per 1 g of dry weight of flower. 

The metabolite contents in the inflorescences were determined from the data for ligulate and tubular flowers. To do this, the metabolite content in the ligulate and tubular flowers was recalculated by taking into account their proportions in the inflorescences and then combining them. The results are expressed in relative units per 1 g of inflorescences. 

### 2.7. Reagents

LiChrosolv^®^ acetonitrile for UPLC-HRMS analysis was purchased from Merck KGaA (Darmstadt, Germany), analytical-grade formic acid from Sigma-Aldrich (Steinheim, Germany), methanol (99.5%, *vol*/*vol*) from Primalco (Rajamäki, Finland) and acetone from VWR Chemicals (EC). Pure water was obtained using an Elgastat UHQ-PS purification system (Elga, Kaarst, Germany). Lidocaine and (1*R*)-(-)-10-camphorsulfonic acid were purchased from Sigma-Aldrich (Steinheim, Germany).

## 3. Results

### 3.1. UPLC-PDA-HRMS Analysis of Metabolome 

**Figure 2:**
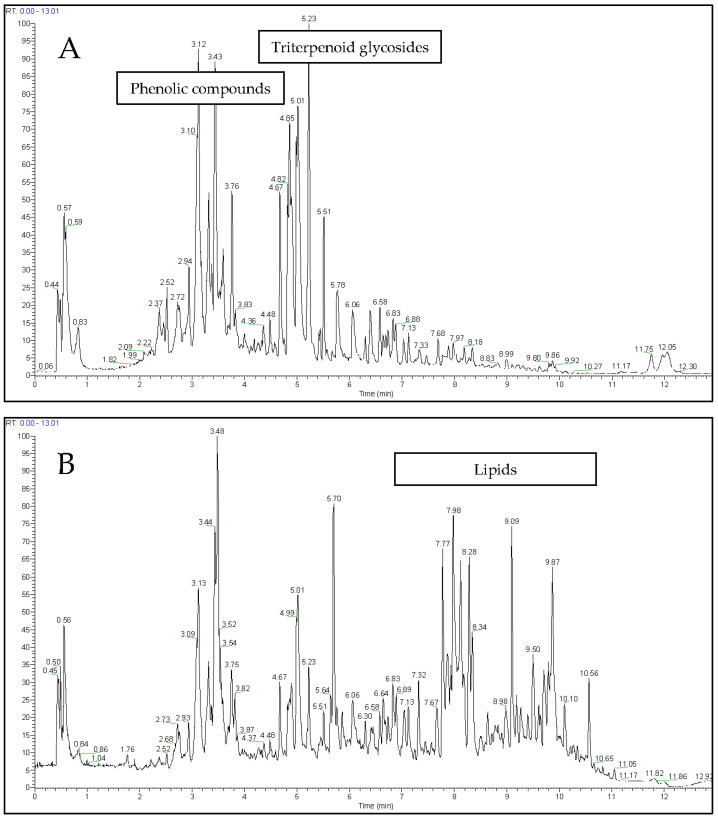
UPLC-HRMS profiles of metabolites in the master sample of extract of *Calendula officinalis* flowers registered in negative (**A**) or positive (**B**) modes.

Ligulate and tubular flowers of *C. officinalis* were extracted with 80% methanol and analysed using UPLC-PDA-HRMS in negative and positive ionisation modes (Figure 2A,B). Comparison of the MS profiles of the master sample under different ionisation modes showed that phenolic compounds and triterpenoid glycosides were more efficiently recorded as negative ions (Figure 2A). In contrast, lipids were better analysed as positive ions (Figure 2B). The total number of negative *m*/*z* ions found in extracts of *C. officinalis* flowers was 18,467, and for positive ions, the number was 25,593.

**Figure 3:**
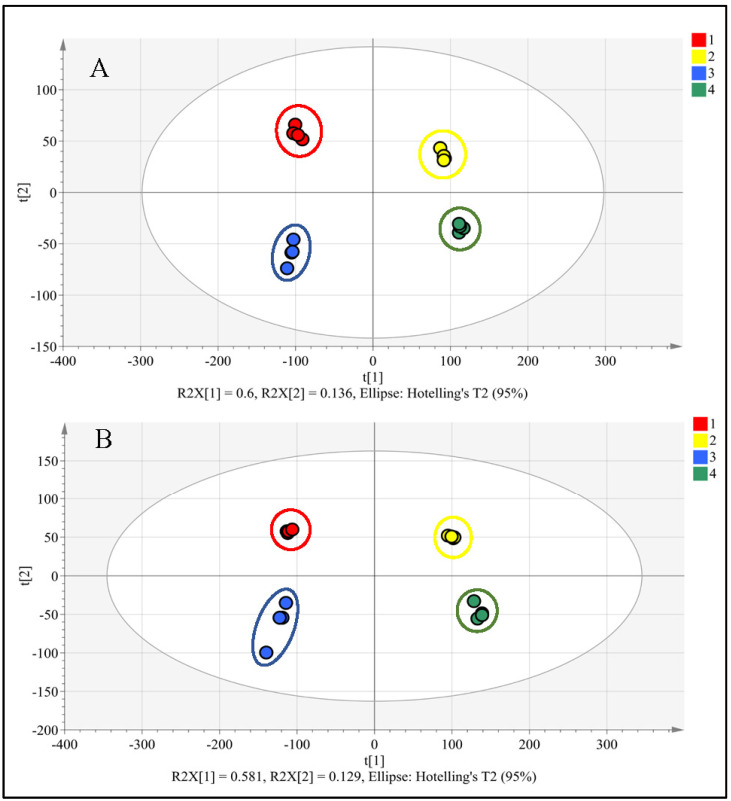
Visualisation of differences in the metabolome of ligulate and tubular flowers of two cultivars of *Calendula officinalis* by the PCA method. (**A**) MS registration of negative ions used for the analysis of phenolic compounds and triterpenoid glycosides (16 samples, 18,467 ions); (**B**) MS registration of positive ions used for polar lipids analysis (16 samples, 25,593 ions). Groups of samples: 1. cultivar ‘Paradise Garden’, ligulate flowers; 2. cultivar ‘Paradise Garden’, tubular flowers; 3. cultivar ‘Golden Sea’, ligulate flowers; 4. cultivar ‘Golden Sea’, tubular flowers.

The PCA model of the complete metabolomic database effectively distinguished four groups of samples belonging to ligulate and tubular flowers of two cultivars of *C. officinalis* (Figure 3). The separation between flower samples was observed along the first principal component (t[1]), which accounted for 60% and 58% of the variance in negative and positive ions in the database, respectively (Figure 3). The second principal component (t[2]) separated the samples of GS and PG cultivars, explaining 14% and 13%, respectively.

However, as the reliability of PCA results is highly dependent on the biological variability of the plants, the OPLS method was used in the next step [33]. The OPLS models for ligulate versus tubular flowers were evaluated using CV-ANOVA. Both models, for negative and positive ions, were found to be highly significant (Table 1). The models for GS versus PG were also significant, but less pronounced than the flower models. The significance of all models was supported by high values of R2 > 0.9 and Q2 > 0.7.

Fifty-eight major metabolites were selected from the analysis of the mass spectrometry data. These compounds include phenolic compounds, triterpenoid glycosides and lipids. No differences in the composition of these metabolites were found between the different flower samples. The high content of these metabolites in the flowers may be due to the pharmacological properties of *C. officinalis*.

### 3.2. Characterisation of Phenolic Compounds

A characteristic feature of phenolic compounds is their ability to absorb light in the UV region of the spectrum that is often used for their registration and preliminary structural characterisation. On the basis of UV spectral data alone, 15 major phenolic compounds were tentatively classified as flavonoids and derivatives of caffeic or *p*-coumaric acids (Table 2; Figure S1).

Compounds P1, P2 and P3 had UV spectra characteristic of caffeic acid derivatives with an absorption maximum in the 325–327 nm region and the same monoisotopic mass of 354.0947 Da (Table 2). Examination of the MS/MS spectra of the deprotonated [M-H]^−^ ion revealed fragments of *m*/*z* 191.0556 [quinic acid-H]^−^ and *m*/*z* 179.0339 [caffeic acid-H]^−^, which are characteristic of caffeoylquinic acids (Table 2). Based on the retention time of caffeoylquinic acid standards [44] and the presence of diagnostic ions in the MS/MS spectrum [34,44], compounds P1, P2 and P3 were identified as 5-, 3- and 4-*O*-caffeoylquinic acids or neochlorogenic, chlorogenic and cryptochlorogenic acids, respectively (Table 2) [5,28].

Compound P12 also exhibited a UV spectrum characteristic of caffeoylquinic acids, but the monoisotopic mass value was 516.1262 Da (Table 2). The MS/MS analysis data of the parent ion showed the presence of fragments *m*/*z* 179.0336 [caffeic acid-H]^−^, *m*/*z* 191.0557 [quinic acid-H]^−^ and *m*/*z* 353.0874 [M-caffeoyl unit]^−^ (Table 2). On this basis, compound P12 was identified as 3,5-dicaffeoylquinic acid [28].

The UV spectra of compounds P4, P5, P8 and P9 showed two absorption maxima in the regions of 254–255 nm and 351–355 nm with a small shoulder at 270 nm, which are characteristic of flavonoids (Table 2). The monoisotopic mass values of these compounds of 756.2108, 610.1531, 464.0950 and 610.1531 Da and the presence of the diagnostic ion *m*/*z* 301.0343 [quercetin-H]^−^ in the MS/MS spectra indicate that these phenolic compounds are quercetin glycosides (Table 2). By comparing the MS of the compounds with data from MS databases, compounds P4, P5, P8 and P9 were identified as quercetin 3-*O*-rutinosyl-rhamnoside, quercetin 3-*O*-β-D-rutinoside (rutin), quercetin 3-*O*-glucoside (isoquercetin) and quercetin 3-*O*-rhamnosyl-glucoside, respectively (Table 2) [5,40,41].

**Table 2:** UPLC-PDA-Q Exactive Orbitrap-HRMS/MS characterisation of phenolic compounds in the extract from *Calendula officinalis* flowers, * shoulder.

Code	RT	UV Maxima	[M-H]^−^	MS/MS	Mass	Chemical	Error	Metabolite
	(min)	λ (nm)	(*m*/*z*)	Fragments (*m*/*z*)	(Da)	Formula	(ppm)	Characterisation
P1	2.37	323	353.0875	179.0339, 191.0556	354.0947	C_16_H_18_O_9_	−1.1	5-*O*-Caffeoylquinic acid [5,28]
P2	2.52	310sh, 326	353.0874	179.0336, 191.0556	354.0946	C_16_H_18_O_9_	−1.4	3-*O*-Caffeoylquinic acid [5,28]
P3	2.72	310sh *, 326	353.0875	179.0340, 191.0555	354.0947	C_16_H_18_O_9_	−1.1	4-*O*-Caffeoylquinic acid [5,28]
P4	2.94	255, 270sh, 354	755.2036	301.0344, 271.0243	756.2108	C_33_H_40_O_20_	−0.7	Quercetin-3-*O*-rutinosyl-rhamnoside [5,40,41]
P5	3.10	254, 270sh, 355	609.1459	301.0342, 271.0243	610.1531	C_27_H_30_O_16_	−0.5	Quercetin-3-*O*-β-D-rutinoside [5,40,41]
P6	3.12	254, 270sh, 356	769.2185	315.0505, 460.1011	770.2257	C_34_H_42_O_20_	−1.6	Isorhamnetin-3-*O*-rutinosyl-rhamnoside [5,41]
P7	3.25	263, 346	593.1512	285.0399, 431.0977	594.1584	C_27_H_30_O_15_	−0.1	Kaempferol-3-*O*-rutinoside [5,45]
P8	3.29	254, 270sh, 355	463.0878	301.0348, 271.0276	464.0950	C_21_H_20_O_12_	−1.0	Quercetin-3-*O*-glucoside [5,40,41]
P9	3.37	254, 270sh, 351	609.1459	301.0343, 271.0244	610.1531	C_27_H_30_O_16_	−0.5	Quercetin-3-*O*-rhamnosyl-glucoside [5,40,41]
P10	3.43	254, 270sh, 356	623.1613	315.0491, 299.0191	624.1685	C_28_H_32_O_16_	−0.9	Isorhamnetin-3-*O*-rutinoside [5,41]
P11	3.48	254, 270sh, 354	623.1612	315.0491, 299.0191	624.1684	C_28_H_32_O_16_	−0.9	Isorhamnetin 3-*O*-rhamnopyranosyl-glucopyranoside [5,41]
P12	3.59	300sh, 328	515.119	179.0336, 191.0557, 353.0874	516.1262	C_25_H_24_O_12_	−1.1	3,5-Di-*O*-caffeoylquinic acid [28]
P13	3.76	299, 308	639.3185	119.0497, 519.2606	640.3257	C_37_H_44_N_4_O_6_	−0.6	Tris-trans-*p*-coumaroyl-spermine
P14	3.83	254, 265sh, 354	563.1036	315.0506, 299.0195	564.1108	C_25_H_24_O_15_	−1.3	Isorhamnetin-malonyl-hexoside
P15	4.82	298, 308	785.3546	119.0495, 639.3183	786.3618	C_46_H_50_N_4_O_8_	−1.4	Tetra-trans-*p*-coumaroyl-spermine

On the basis of UV spectra, compounds P6, P10, P11 and P14 were also classified as flavonoids (Table 2). The monoisotopic masses of these flavonoids were 770.2257, 624.1685, 624.1684 and 564.1108 Da, respectively. The presence of the diagnostic ion *m*/*z* 315.0506 [isorhamnetin-H]^−^ in the MS/MS spectra indicates that these compounds are isorhamnetin glycosides (Table 2). Comparing the MS of the compounds with data from MS databases, flavonoids P6, P10 and P11 were identified as isorhamnetin-3-*O*-rutinosyl-rhamnoside, isorhamnetin-3-*O*-rutinoside (narcissin) and isorhamnetin 3-*O*-rhamnopyranosyl-glucopyranoside (calendoflavoside), respectively (Table 2) [5,41]. Flavonoid P14 was identified as isorhamnetin malonyl hexoside (Table 2).

Compound P7 had a UV spectrum with absorption maxima at 263 and 346 nm, characteristic of kaempferol glycosides (Table 2). Based on the monoisotopic mass of 594.1584 Da and the diagnostic MS/MS ion *m*/*z* 285.0399 [kaempferol-H]^−^, compound P7 was identified as kaempferol-3-*O*-rutinoside, which has been found in *C. arvensis* inflorescences [5,45].

Compounds P13 and P15 had UV spectra with absorption maxima at 299 and 308 nm, characteristic of p-coumaric acid, and monoisotopic masses of 640.3257 and 786.3618 Da, respectively (Table 2). MS database searches identified these compounds as tris-trans-*p*-coumaroyl-spermine (P13) and tetra-trans-*p*-coumaroyl-spermine (P15) (Table 2). The identifications were confirmed by the presence of the ion *m*/*z* 639.3185 [M-p-coumaric acid]^−^ with MS/MS fragmentation of the parent ion *m*/*z* 785.3546 [M-H]^−^ of compound P15 (Table 2). Both of these compounds have been found in the flowers of plants belonging to the Asteraceae family, but not in the flowers of *C. officinalis* [46].

Thus, 15 major phenolic compounds were found in the ligulate and tubular flowers of *C. officinalis*. Derivatives of caffeic acid and p-coumaric acid were identified, as well as glycosides of quercetin, kaempferol and isorhamnetin. Isorhamnetin malonyl hexoside, kaempferol 3-*O*-rutinoside, tris-trans-*p*-coumaroyl-spermine and tetra-trans-*p*-coumaroyl-spermine were identified in the flowers of *C. officinalis* for the first time.

### 3.3. Characterisation of Triterpenoid Glycosides

MS analysis of the metabolites of *C. officinalis* flowers revealed seven triterpenoid glycosides with retention times ranging from 4.5 to 6.5 min (Figure 2A). Compounds characteristic of *C. officinalis*, such as esters of triterpenoids and fatty acids, were not found in the 80% methanol extract, because non-polar organic solvents must be used to extract these lipophilic compounds.

Compound T1 had a monoisotopic mass of 1118.5514 Da and a chemical formula of C_54_H_86_O_24_ (Table 3). MS/MS of the parent ion *m*/*z* 1117.5436 [M-H]^−^ showed the presence of a fragment *m*/*z* 455.3543 belonging to the oleanolic acid ion [oleanolic acid-H]^−^ (Table 3). As a result, compound T1 was identified as oleanolic acid tetraglycoside or calendulaglycoside A [3,42,47].

The next two compounds, T2 and T5, had the same monoisotopic mass values of 956.4971 Da and the chemical formula C_48_H_76_O_19_ (Table 3). The presence of the *m*/*z* 455.3524 fragment in the MS/MS spectrum indicates that these isomers are the oleanolic acid triglycosides, calendulaglycoside B and calendulaglycoside C [5,45]. T3 and T6 with the same mass of 794.4455 Da and the formula C_42_H_66_O_14_ are isomers of the oleanolic acid diglycoside: calendulosides G and F [3,42,47].

Compound T4, with a monoisotopic mass of 836.4559 Da, was tentatively identified as the acetyloleanolic acid glucuronide hexoside (Table 3). This is supported by the fact that the major MS/MS fragments of the parent ion *m*/*z* 835.4481 [M-H]^−^ were the ions *m*/*z* 497.3636 [acetyloleanolic acid-H]^−^, *m*/*z* 455.3521 [oleanolic acid-H]^−^ and *m*/*z* 793.4368 [M-acetyl-H]^−^ (Table 3). The acetyloleanolic acid glucuronide hexoside was found for the first time in the flowers of *C. officinalis*.

Compound T7 with mass 632.3921 Da and chemical formula C_36_H_56_O_9_ was identified as oleanolic acid glucuronide or calenduloside E (Table 3), which is the precursor in the synthesis of di-, tri- and tetra-glycosides of oleanolic acid and acetyoleanolic acid glucuronide hexoside [3,47].

### 3.4. Characterisation of Lipids

When the extracts of the flowers were analysed by means of UPLC-PDA-HRMS in the positive ionisation mode, 36 major compounds were detected. The compounds in this group belong to the relatively polar lipids, which are soluble in the polar 80% methanol [48]. Mass spectrometry allows accurate calculation of the lipid chemical formula, but it is very difficult to determine the position of the double bonds and the structural isomers. For this reason, lipids with the same mass were tentatively characterised as isomers (Table 4 and Table S2).

Compound L1 had a monoisotopic mass of 328.2247 Da and a chemical formula of C_18_H_32_O_5_ (Table 4 and Table S2). MS/MS of the parent ion *m*/*z* 329.2325 [M+H]^+^ showed the presence of fragments *m*/*z* 311.2210 [M-H_2_O+H]^+^, 293.2106 [M-2H_2_O+H]^+^ and 275.2004 [M-3H_2_O+H]^+^. As a result, compound L1 was identified as trihydroxyoctadecadienoic acid (Table 4 and Table S2). Two compounds L4 and L5 with the same mass of 294.2198 Da and formula C_18_H_30_O_3_ also belong to the group of octadecadienoic acids and were identified as isomers of oxooctadecadienoic acid (Table 4 and Table S2).

Four compounds L2, L6, L9 and L16 with identical monoisotopic mass values of 278.2241 Da and chemical formula C_18_H_30_O_2_ were characterised as isomers of octadecatrienoic acid (Table 4 and Table S2). Previously, three of these isomers were identified as α- and β-calendic acids and α-linolenic acid [19,20]. Three octadecatrienoic acid derivatives (L10, L12 and L15) were also detected (Table 4 and Table S2). MS and MS/MS database searches identified them as hydroxyoctadecatrienoyl-carnitine, octadecatrienoyl-sn-glycerol and octadecatrienoic acid 2,3-bis(acetyloxy)propyl ester, respectively (Table 4 and Table S2).

In addition to C18-polyunsaturated fatty acids, C23 and C28 fatty acids and their derivatives were detected in *C. officinalis* flowers. Compounds L17 and L21 with masses of 454.4017 and 482.4336 Da were identified as octacosanedioic acid and dimethyloctacosanedioic acid, respectively; L19 and L25 with the same mass of 452.3862 Da were identified as two isomers of dioxooctacosanoic acid, and compound L18 with a mass of 348.3022 Da and chemical formula C_23_H_40_O_2_ was identified as tricosatrienoic acid (Table 4 and Table S2).

The two compounds L7 and L8 had the same mass of 336.2662 Da and the chemical formula C_21_H_36_O_3_ (Table 4 and Table S2). Based on MS/MS fragmentation of the parent ion [M+H]^+^, they were identified as isomers of dimethyl-pentyl-furandecanoic acid, which is a heterocyclic fatty acid containing a furan ring in the molecule (Table 4 and Table S2). Another compound L11 with a mass of 364.2971 Da also belongs to the group of furan fatty acids and was identified as dimethyl-pentyl-furandodecanoic acid (Table 4 and Table S2).

Compounds L23 and L28 had the same monoisotopic mass of 480.4179 Da and the chemical formula C_30_H_56_O_4_. According to the results of MS/MS fragmentation of the parent ion [M+H]^+^, they were identified as isomers of the ditridecyl ester of butenedioic acid (Table 4 and Table S1).

Compound L3 with a monoisotopic mass of 315.2771 Da and chemical formula C_18_H_37_NO_3_ was identified as a dehydrophytosphingosine belonging to the class of amino alcohols [49]. The identification result was confirmed by MS/MS fragmentation data of the parent ion [M+H]^+^ (Table 4 and Table S1).

Compound L27 with a monoisotopic mass of 757.5604 Da and chemical formula C_42_H_80_NO_8_P containing nitrogen and phosphorus atoms was identified as oxidised phosphatidylcholine (Table 4 and Table S1). This compound belongs to the class of glycerophospholipids in which glycerol is substituted by a phosphorylcholine moiety and at least one of the fatty acyl chains has undergone oxidation [50].

Two compounds, L20 and L24, with monoisotopic masses of 302.2604 and 330.2919 Da, respectively, were identified as pentadecenyl-phenol and heptadecenyl-phenol (Table 4 and Table S1). Both compounds belong to the phenolic lipid class or cardanols [51]. Two other compounds, L22 and L26, with monoisotopic masses of 406.3080 and 472.3554 Da, respectively, were also assigned to the phenolic lipid class (Table 4 and Table S1). The identification results were based on the presence of the *m*/*z* fragment 179.1063 [hydroxyphenylpentanone+H]^+^ in the MS/MS spectrum of both compounds.

Some lipids were only tentatively characterised as compounds with certain functional groups in the molecule. For example, compounds L13 and L14 were classified as aminolipids (Table 4 and Table S1). The remaining lipids (L29–L36) could not be identified despite detailed MS and MS/MS analyses and database searches. 

### 3.5. Comparison of Metabolites of Ligulate and Tubular Flowers

**Figure 4:**
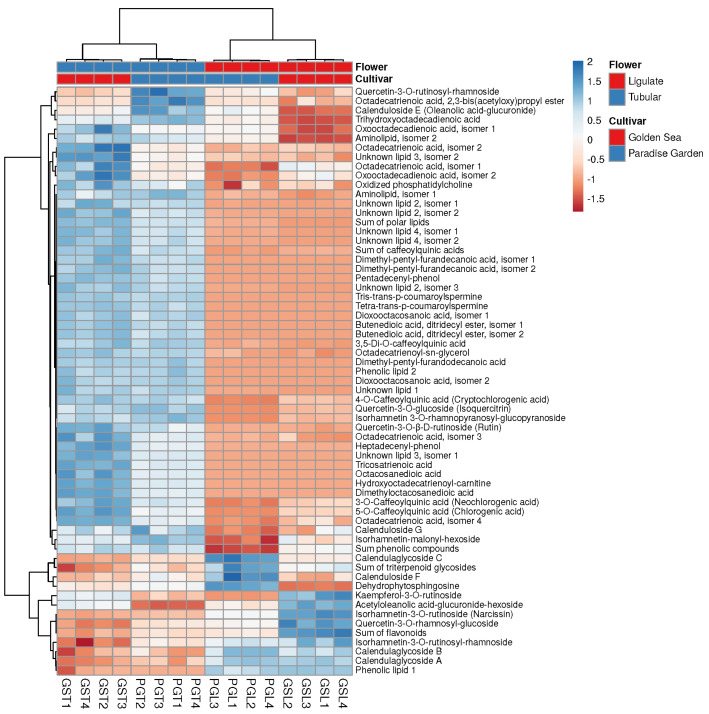
Heatmap of major metabolites showing differences between ligulate and tubular flowers of two cultivars ‘Paradise Garden’ (PG) and ‘Golden Sea’ (GS). Heatmap data matrix visualises the values in the cells using a color gradient, which gives an overview of the largest (blue color) and smallest (brown color) values in the matrix. Rows are centered; unit variance scaling is applied to rows. Both rows and columns are clustered using correlation distance and average linkage; 63 rows, 16 columns.

The ligulate and tubular flowers had a similar composition of compounds, but differed in their content (Figure 4, Table 5). The comparison of phenolic compound content between the two types of flowers showed that the tubular flowers of both *C. officinalis* cultivars contained 2 to 5 times more caffeoylquinic acids than the ligulate flowers (Table 5). This study found that the five flavonoids—quercetin-3-*O*-rutinosyl-rhamnoside, quercetin-3-*O*-β-D-rutinoside (rutin), kaempferol-3-*O*-rutinoside, quercetin-3-*O*-glucoside (isoquercitrin) and isorhamnetin 3-*O*-rhamnopyranosyl-glucopyranoside—followed the same trend (Table 5). In contrast, ligulate flowers had higher levels of isorhamnetin-3-*O*-rutinosyl-rhamnoside, quercetin-3-*O*-rhamnosyl-glucoside and isorhamnetin-3-*O*-rutinoside (narcissin) than tubular flowers (Table 5). These results are consistent with those reported by Olennikov and Kashchenko [28].

Tris-trans-*p*-coumaroyl-spermine and tetra-trans-*p*-coumaroyl-spermine contents were found to differ significantly between flowers. In both cultivars, the content of these *p*-coumaric acid derivatives was 82 to 88 times higher in the tubular flowers than in the ligulate flowers (Table 5). However, differences in total phenolic content between flowers were only observed for the PG cultivar.

When comparing the triterpenoid glycoside content in the flowers, it was discovered that the ligulate flowers of both cultivars contained approximately 1.3–2.2 times more calendulaglycosides A, B and C; calenduloside F; and acetyloleanolic acid glucuronide hexoside than the tubular flowers (Table 5). However, the levels of calendulosides E and G in ligulate flowers were found to be 1.4–2.0 times lower. Table 5 shows that both cultivars of *C. officinalis* have a higher total content of triterpenoid glycosides in their ligulate flowers compared to the tubular flowers.

The lipids showed the most significant differences between the two flower types (Table 5). In the tubular flowers of GS and PG, a higher content was found for 31 and 34 of the 36 lipids, respectively. The content of certain lipids, such as dimethylpentylfurandecanoic acid isomer 2, octacosanedioic acid, tricosatrienoic acid, dioxooctacosanedioic acid isomer 1, dimethyloctacosanedioic acid, pentadecenylphenol and heptadecenylphenol, differed 100-fold or more between flowers. In addition, the total content of lipids in the tubular flowers of both cultivars was about four times higher than that in the ligulate flowers.

### 3.6. Comparison of the Metabolites of Two C. officinalis Cultivars

Initially, a comparison between the two cultivars of *C. officinalis* regarding the content of major metabolites was made for the ligulate and tubular flowers separately. The results of the PCA indicated that the quantitative differences between two cultivars for the same flower were much smaller than those between the ligulate and tubular flowers of the cultivar (Figure 3).

Table S3 shows that the ligulate flowers of the GS contain a larger amount of caffeoylquinic acids and flavonoids compared to the PG cultivar. For the tubular flowers, the differences in the phenolics content between two cultivars were less pronounced, although the GS cultivar also had a higher content of neochlorogenic and chlorogenic acids and less content of calendulaglycoside C and calenduloside E than the PG cultivar.

The analysis revealed significant differences in lipids among the cultivars’ flowers (Table S3). The PG cultivar had a higher total lipid content, including 16 individual compounds, in the ligulate flowers, while the GS cultivar had a higher total lipid content, including 15 individual compounds, in the tubular flowers.

**Figure 5:**
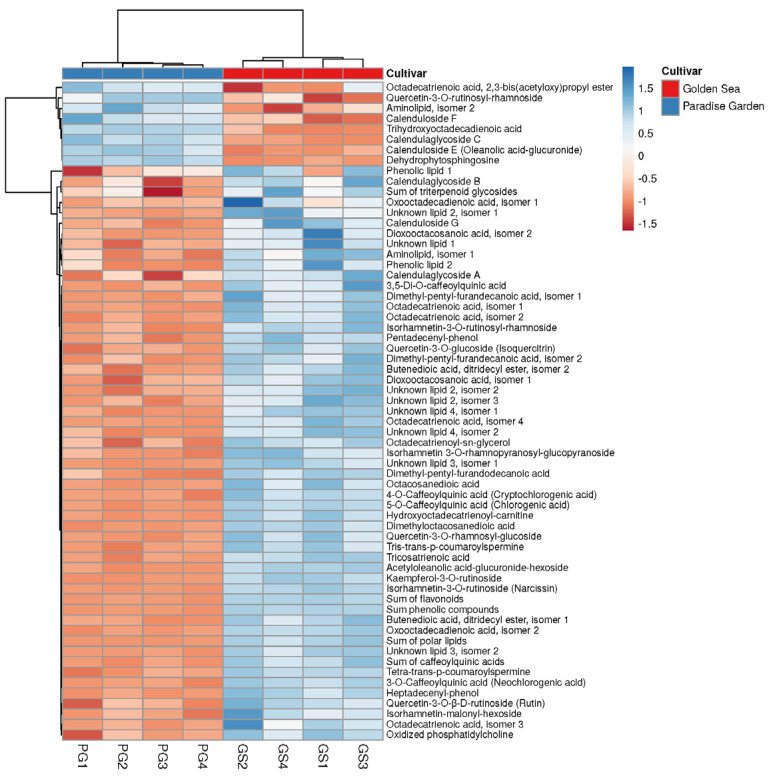
Heatmap of major metabolites showing differences between inflorescences of two cultivars ‘Paradise Garden’ (PG) and ‘Golden Sea’ (GS). Heatmap data matrix visualises the values in the cells using a color gradient which gives an overview of the largest (blue color) and smallest (brown color) values in the matrix. Rows are centered; unit variance scaling is applied to rows. Both rows and columns are clustered using correlation distance and average linkage; 63 rows, 8 columns.

The obtained results enable a comparison of *C. officinalis* cultivars based solely on the metabolite content in ligulate and tubular flowers. However, it is important to note that the primary raw material for medicinal preparations derived from *C. officinalis* are inflorescences [25], where the proportion of lingulate and tubular flowers may vary depending on the cultivar [26,31]. For example, GS has 35% ligulate and 31% tubular flowers, whereas PG has 36% ligulate and 22% tubular flowers (Table S1). Therefore, we compared the studied cultivars based on the metabolite content of their inflorescences (Figure 5, Table 6). To achieve this, we recalculated the metabolite content in ligulate and tubular flowers, considering their proportions in the inflorescence, and then combined them.

This study found that 45 out of 58 metabolites had a higher content in the inflorescences of GS compared to PG (Figure 5, Table 6). Among the phenolic compounds, GS inflorescences had a higher content of 13 out of 15 metabolites. As a result, the total content of phenolic compounds, flavonoids and caffeoylquinic acids was 1.3 to 1.5 times higher in GS. Out of the 36 polar lipids, 30 were found to have higher levels in GS inflorescences. The exceptions were trihydroxyoctadecadienoic acid and dehydrophytosphingosine, which were 1.3 and 1.9 times lower in GS than in PG, respectively. When *C. officinalis* cultivars were compared in terms of triterpenoid glycoside content, it was found that GS inflorescences had higher levels of calenduloside G and acetyloleanolic acid glucuronide hexoside, whereas PG inflorescences had higher levels of calendulaglycoside C and calendulosides F and E (Figure 5, Table 6). Therefore, no differences in total triterpenoid glycoside content were observed between the inflorescences of the two *C. officinalis* cultivars.

By analysing the metabolite content of the ligulate and tubular flowers that form the inflorescences, we were able to understand which flower contributed most to the inflorescence metabolome (Figure 5, Table 6). For example, the higher lipid content in GS inflorescences was dependent on their content in tubular flowers. However, the content of most phenolic compounds in GS was equally determined by metabolites from both flower types. In the group of triterpenoids, both flowers also had a higher content of calenduloside G and acetyloleanolic acid glucuronide hexoside in the inflorescences of the GS cultivar, and only the ligulate flowers had a higher content of calendulaglycoside C and calendulosides F and E in the inflorescences of the PG cultivar (Figure 5, Table 6).

## 4. Discussion

In this study, UPLC-PDA-HRMS-based metabolomics was used to characterise the major compounds from the ligulate and tubular flowers of two cultivars of *C. officinalis* that may be responsible for the medicinal properties of the plant. As a result, in addition to the well-known caffeoylquinic acids, flavonoids and triterpenoid glycosides, a number of previously unknown metabolites have been identified in the flowers of *C. officinalis*. Of particular interest among the first discovered metabolites were tri-trans-*p*-coumaroyl-spermine and tetra-trans-*p*-coumaroyl-spermine, which belong to the phenolamide class.

Phenolamides are a diverse class of plant secondary metabolites formed by conjugating hydroxycinnamic acids (*p*-coumaric, caffeic, ferulic and sinapic acids) with aromatic monoamines (tyramine, tryptamine and dopamine) or aliphatic polyamines (putrescine, spermidine, spermine and agmatine) [52,53]. They exhibit a broad range of pharmaceutical activities, including anti-inflammatory, antimicrobial and anticancer properties. Phenolamides are known to protect human health against metabolic syndrome, cardiovascular diseases and neurodegenerative diseases [54,55]. Tetra-trans-*p*-coumaroyl-spermine has been used successfully in the treatment of depression and anxiety [56,57]. According to [58], phenolamides have a significantly higher antioxidant activity than flavonoids, which are generally considered to be the major antioxidants in *C. officinalis*. Therefore, the presence of the biologically active phenolamides in the flowers of *C. officinalis* may significantly expand the use of medicinal preparations derived from this plant.

This study compared the content of metabolites in ligulate and tubular flowers of *C. officinalis*. Among the phenolic compounds, the most significant differences between the two flower types were found in the content of phenolamides. Functionally male tubular flowers contained very high levels of tris-trans-*p*-coumaroyl-spermine and tetra-trans-*p*-coumaroyl-spermine, which play an important role in pollen development and germination [54,58,59,60,61]. They are also involved in plant development, the induction of flowering and the enhancement in resistance to biotic and abiotic stresses [54,58,59,60,61].

In addition to phenolamides, the tubular flowers also contain higher levels of caffeoylquinic acids, which are also derivatives of *p*-coumaric acid. Therefore, in contrast to ligulate flowers, the metabolism of tubular flowers is characterised by a higher activity of the pathway for the conversion of *p*-coumaric acid into caffeoylquinic acids and phenolamides.

Compared to tubular flowers, the ligulate flowers of both cultivars contained higher levels of triterpenoid glycosides. This finding is consistent with previous research on the triterpenoid ester content of ligulate flowers of *C. officinalis* [30]. The authors suggest that cultivars with a high number of ligulate flowers should be selected to increase the content of biologically active triterpenoids in marigold inflorescences and improve their medicinal properties [30].

The most significant differences between ligulate and tubular flowers were found in the lipid group. The higher content of almost all lipids in tubular flowers than in ligulate flowers suggests that these metabolites play a crucial role in the development of male tubular flowers of *C. officinalis* [62,63,64,65].

Calendic acids are known pharmacologically active lipids that were previously found in *C. officinalis* seeds [22,23,24]. Ligulate and tubular flowers both contain four octadecatrienoic acid isomers and three of their derivatives, including hydroxyoctadecatrienoyl-carnitine, octadecatrienoyl-sn-glycerol and octadecatrienoic acid 2,3-bis(acetyloxy)propyl ester. The content of octadecatrienoic acids and their derivatives in tubular flowers was only slightly higher than in ligulate flowers.

Octadecadienoic acid is the biosynthetic precursor of octadecatrienoic acids in plants [66,67]. The free form of octadecadienoic acid was not detected in the flowers of *C. officinalis*. However, its derivatives, including trihydroxyoctadecadienoic acid and two isomers of oxooctadecadienoic acid, were present.

Thus, the comparative metabolomics of ligulate and tubular flowers showed significant differences in the content of the major metabolites of *C. officinalis* flowers. Among the most pronounced differences are a significantly higher content of phenolamides, caffeoylquinic acids and most lipids in tubular flowers, and a higher content of triterpenoid glycosides in ligulate flowers.

The metabolome differences between the identical flowers of the two *C. officinalis* cultivars, GS and PG, were found to be minimal. However, since the primary raw material for medicinal products derived from *C. officinalis* are the inflorescences [25], it was more appropriate to compare the metabolite content of the two cultivars using data for inflorescences.

The results showed that the inflorescences of the GS contained 30% more phenolic compounds and 50% more lipids than those of the PG. The reasons why the inflorescences of the GS had the highest amount of biologically active compounds were due to the higher content of these metabolites in the tubular flowers and the 35% higher proportion of tubular flowers in the inflorescences.

Therefore, the results obtained extend our understanding of the chemical factors influencing the medicinal properties of *C. officinalis* and provide insight into the metabolomic characteristics of the ligulate and tubular flowers. Furthermore, they indicate that the metabolome of *C. officinalis* inflorescences is also dependent on the proportions of ligulate and tubular flowers in the inflorescences.

## 5. Conclusions

This study found significant metabolomic differences between the ligulate and tubular flowers of two cultivars of *C. officinalis*, GS and PG. Tubular flowers had higher levels of lipids, phenolamides and caffeoylquinic acids but lower levels of triterpenoid glycosides compared to ligulate flowers. It was also shown that the inflorescence of the GS contained significantly more lipids and phenolic compounds than the PG. These differences between cultivars may be due to the different proportions of ligulate and tubular flowers in their inflorescences. The inflorescences of GS had a 35% higher proportion of tubular flowers than those of PG. Based on these results, we suggest that the inflorescences of GS could be a better raw material for the preparation of medicinal products where the pharmaceutical activity is determined by phenolic compounds or lipids. However, to accurately evaluate the phytochemical and medicinal properties of the cultivars, further investigation of other biologically active compounds such as carotenoids, lipophilic triterpenoids and non-polar lipids is required. It is also essential to investigate the relationship between the content of individual metabolites and the various pharmaceutical activities of the extracts. Thus, this study is our first attempt to understand which components of the *C. officinalis* metabolome may be responsible for the multiple medicinal properties of the plant.

## Figures and Tables

**Table 1:** Evaluating the statistical significance of OPLS models with ANOVA of cross-validated predictive residuals. Abbreviations: LF, ligulate flowers; TF, tubular flowers; PG, ‘Paradise Garden’; GS, ‘Golden Sea’.

Cultivar, Flowers	Compared	Negative Ions		Positive Ions	
	Groups	F	*p*	F	*p*
‘Paradise Garden’	LF vs. TF	524.3	1.55 × 10^−6^	601.4	1.10 × 10^−6^
‘Golden Sea’	LF vs. TF	527.7	1.53 × 10^−6^	675.6	8.25 × 10^−7^
Ligulate flowers	PG vs. GS	17.1	0.036	22.7	0.014
Tubular flowers	PG vs. GS	29.2	0.004	45.4	0.002

**Table 3:** UPLC-PDA-Q Exactive Orbitrap-HRMS/MS characterisation of triterpenoid glycosides in the extract from *Calendula officinalis* flowers.

Code	RT	[M-H]^−^	MS/MS	Mass	Chemical	Error	Metabolite
	(min)	(*m*/*z*)	Fragments (*m*/*z*)	(Da)	Formula	(ppm)	Characterisation
T1	4.85	1117.5436	455.3543, 971.4818	1118.5514	C_54_H_86_O_24_	0.4	Calendulaglycoside A [3,42,47]
T2	5.01	955.4893	455.3524, 793.4368	956.4971	C_48_H_76_O_19_	−0.9	Calendulaglycoside B [5,45]
T3	5.23	793.4377	455.3521, 631.3848	794.4455	C_42_H_66_O_14_	0.3	Calenduloside G [3,42,47]
T4	5.51	835.4481	497.3636, 455.3521, 793.4368	836.4559	C_44_H_68_O_15_	0.1	Acetyloleanolic acid-glucuronide-hexoside
T5	5.78	955.4913	455.3524, 793.4368	956.4991	C_48_H_76_O_19_	1.1	Calendulaglycoside C [5,45]
T6	6.06	793.4379	455.3524, 631.3846	794.4457	C_42_H_66_O_14_	0.6	Calenduloside F [3,42,47]
T7	6.34	631.3843	455.3515	632.3921	C_36_H_56_O_9_	−0.5	Calenduloside E (Oleanolic acid-glucuronide) [3,47]

**Table 4:** UPLC-PDA-Q Exactive Orbitrap-HRMS/MS characterisation of lipids in the extract from *Calendula officinalis* flowers.

Code	RT	[M+H]^+^	MS/MS	Mass	Chemical	Error	Metabolite
	(min)	(*m*/*z*)	Fragments (*m*/*z*)	(Da)	Formula	(ppm)	Characterisation
L1	4.67	329.2325	311.2210, 293.2106, 275.2004	328.2247	C_18_H_32_O_5_	−0.8	Trihydroxyoctadecadienoic acid
L2	4.72	279.2319	261.2213, 243.2108	278.2241	C_18_H_30_O_2_	−1.7	Octadecatrienoic acid, isomer 1 [19,20]
L3	5.70	316.2844	298.2736, 280.2826	315.2771	C_18_H_37_NO_3_	−0.6	Dehydrophytosphingosine
L4	6.45	295.2274	277.2155, 259.2052	294.2198	C_18_H_30_O_3_	1.0	Oxooctadecadienoic acid, isomer 1
L5	6.58	295.2274	277.2155, 259.2052	294.2198	C_18_H_30_O_3_	1.0	Oxooctadecadienoic acid, isomer 2
L6	6.64	279.2317	261.2213, 243.2108	278.2244	C_18_H_30_O_2_	−0.5	Octadecatrienoic acid, isomer 2 [19,20]
L7	6.83	337.2738	319.2632, 301.2518, 247.2953	336.2662	C_21_H_36_O_3_	−0.8	Dimethyl-pentyl-furandecanoic acid, isomer 1
L8	6.93	337.2738	319.2632, 301.2518, 247.2059	336.2662	C_21_H_36_O_3_	−0.8	Dimethyl-pentyl-furandecanoic acid, isomer 2
L9	7.11	279.2316	261.2213, 243.2108	278.2243	C_18_H_30_O_2_	−1.2	Octadecatrienoic acid, isomer 3 [19,20]
L10	7.13	438.322	379.2468	437.3147	C_25_H_43_NO_5_	1.4	Hydroxyoctadecatrienoyl-carnitine
L11	7.32	365.3046	347.2946, 329.2472	364.2971	C_23_H_40_O_3_	−1.8	Dimethyl-pentyl-furandodecanoic acid
L12	7.41	353.2686	335.2579, 261.2210	352.2613	C_21_H_36_O_4_	−0.2	Octadecatrienoyl-sn-glycerol
L13	7.51	473.2395	225.1846, 207.1740, 189.1634	472.2322	C_25_H_28_N_8_O_2_	−2.8	Aminolipid, isomer 1
L14	7.62	473.2397	225.1846, 207.1740	472.2324	C_25_H_28_N_8_O_2_	−2.3	Aminolipid, isomer 2
L15	7.67	437.2906	247.1692, 233.1534	436.2833	C_25_H_40_O_6_	1.8	Octadecatrienoic acid, 2,3-bis(acetyloxy)propyl ester
L16	7.77	279.2317	261.2213, 243.2108	278.2244	C_18_H_30_O_2_	−0.8	Octadecatrienoic acid, isomer 4 [19,20]
L17	7.83	455.4093	419.3877, 365.3410	454.4017	C_28_H_54_O_4_	−1.2	Octacosanedioic acid
L18	7.90	349.3095	331.2990, 261.2209	348.3022	C_23_H_40_O_2_	−1.7	Tricosatrienoic acid
L19	8.17	453.3935	435.3820, 349.3094	452.3862	C_28_H_52_O_4_	−0.9	Dioxooctacosanoic acid, isomer 1
L20	8.28	303.268	285.2573, 221.1898	302.2604	C_21_H_34_O	−1.9	Pentadecenyl-phenol
L21	8.34	483.4409	465.4303, 393.3723	482.4336	C_30_H_58_O_4_	0.2	Dimethyloctacosanedioic acid
L22	8.56	407.3153	207.1377, 179.1064, 161.0959	406.308	C_25_H_42_O_4_	−0.7	Phenolic lipid 1
L23	8.98	481.4245	463.4138, 409.3671, 377.3409	480.4179	C_30_H_56_O_4_	0.1	Butenedioic acid, ditridecyl ester, isomer 1
L24	9.09	331.2992	313.2887, 239.2366, 109.1015	330.2919	C_23_H_38_O	−1.1	Heptadecenyl-phenol
L25	9.22	453.3935	435.3821, 349.3094, 295.2630	452.3862	C_28_H_52_O_4_	−0.9	Dioxooctacosanoic acid, isomer 2
L26	9.40	473.3627	273.1848, 247.1324, 245.1532	472.3554	C_30_H_48_O_4_	0.2	Phenolic lipid 2
L27	9.50	758.5676	429.3724, 184.0731	757.5604	C_42_H_80_NO_8_P	−2.4	Oxidised phosphatidylcholine
L28	9.54	481.4247	463.4139, 409.3671, 377.3409	480.4179	C_30_H_56_O_4_	0.1	Butenedioic acid, ditridecyl ester, isomer 2
L29	9.56	465.3934	379.3198, 309.2785, 295.2626	464.3862	C_29_H_52_O_4_	−0.9	Unknown lipid 1
L30	9.58	479.4086	393.3354, 375.3250, 323.2941	478.4025	C_30_H_54_O_4_	0.6	Unknown lipid 2, isomer 1
L31	9.63	429.3723	191.1063, 165.0908	428.365	C_29_H_48_O_2_	−0.9	Unknown lipid 3, isomer 1
L32	9.71	479.4098	393.3354, 375.3250, 323.2940	478.4025	C_30_H_54_O_4_	0.6	Unknown lipid 2, isomer 2
L33	9.84	493.4247	407.3511, 389.3404, 379.3193	492.4174	C_31_H_56_O_4_	−1.0	Unknown lipid 4, isomer 1
L34	9.87	429.3722	191.1068; 165.0909	428.3649	C_29_H_48_O_2_	−1.2	Unknown lipid 3, isomer 2
L35	10.10	479.4096	393.3355, 375.3250, 323.2942	478.4019	C_30_H_54_O_4_	−0.7	Unknown lipid 2, isomer 3
L36	10.56	493.4248	407.3512, 389.3404, 379.3193	492.4175	C_31_H_56_O_4_	−0.8	Unknown lipid 4, isomer 2

**Table 5:** Differences between the ligulate and tubular flowers in the relative content of the metabolites in two cultivars of *Calendula officinalis*, ‘Paradise Garden’ and ‘Golden Sea’.

Metabolite	‘Paradise Garden’			‘Golden Sea’		
	Tubular Flowers/Ligulate Flowers			Tubular Flowers/Ligulate Flowers		
	Ratio ^a^	*t*-Test	Correlation ^c^	Ratio ^a^	*t*-Test	Correlation ^c^
	(fold)	*p* ^b^	*r*	(fold)	*p*	*r*
3-*O*-Caffeoylquinic acid (Neochlorogenic acid)	2.77	***	0.99	2.12	***	0.97
5-*O*-Caffeoylquinic acid (Chlorogenic acid)	5.23	***	0.93	3.21	***	0.96
4-*O*-Caffeoylquinic acid (Cryptochlorogenic acid)	4.33	***	1.00	2.61	***	0.98
Quercetin-3-*O*-rutinosyl-rhamnoside	1.31	***	0.83	1.04	n.s.	0.89
Quercetin-3-*O*-β-D-rutinoside (Rutin)	2.30	***	0.89	3.03	***	0.54
Isorhamnetin-3-*O*-rutinosyl-rhamnoside	−1.10	***	−0.93	−1.38	***	−0.92
Kaempferol-3-*O*-rutinoside	1.36	***	0.90	−1.39	**	−0.82
Quercetin-3-*O*-glucoside (Isoquercitrin)	7.73	***	0.99	3.39	***	0.99
Quercetin-3-*O*-rhamnosyl-glucoside	−1.08	**	−0.80	−1.78	***	−0.99
Isorhamnetin-3-*O*-rutinoside (Narcissin)	−1.49	***	−0.87	−2.45	***	−0.94
Isorhamnetin 3-*O*-rhamnopyranosyl-glucopyranoside	4.70	***	0.99	2.71	***	0.99
3,5-Di-*O*-caffeoylquinic acid	3.87	***	0.99	4.59	***	0.92
Tris-trans-*p*-coumaroyl-spermine	88.08	***	1.00	82.08	***	1.00
Isorhamnetin-malonyl-hexoside	1.92	***	0.99	1.16	*	0.77
Tetra-trans-*p*-coumaroyl-spermine	90.50	***	1.00	92.76	***	1.00
Calendulaglycoside A	−1.45	***	−0.84	−1.62	***	−0.99
Calendulaglycoside B	−1.28	***	−0.95	−1.30	***	−0.95
Calenduloside G	1.37	***	0.87	1.15	n.s.	0.34
Acetyloleanolic acid-glucuronide-hexoside	−1.53	***	−0.93	−1.27	***	−0.50
Calendulaglycoside C	−2.16	***	−0.95	−1.59	***	−0.90
Calenduloside F	−1.41	***	−0.86	−1.01	n.s.	−0.38
Calenduloside E (Oleanolic acid-glucuronide)	1.42	***	0.89	2.01	***	0.87
Trihydroxyoctadecadienoic acid	1.49	***	0.99	2.68	***	1.00
Octadecatrienoic acid, isomer 1	1.45	***	−0.93	1.30	**	0.81
Dehydrophytosphingosine	−1.52	***	−0.94	1.96	***	0.97
Oxooctadecadienoic acid, isomer 1	1.03	n.s.	0.61	1.91	***	0.98
Oxooctadecadienoic acid, isomer 2	1.24	***	0.91	1.29	***	0.82
Octadecatrienoic acid, isomer 2	1.39	***	0.93	2.15	***	0.96
Dimethyl-pentyl-furandecanoic acid, isomer 1	38.35	***	0.99	52.17	***	0.97
Dimethyl-pentyl-furandecanoic acid, isomer 2	384.21	***	0.89	867.96	***	0.95
Octadecatrienoic acid, isomer 3	1.98	***	0.93	2.74	***	0.85
Hydroxyoctadecatrienoyl-carnitine	38.15	***	0.99	78.97	***	1.00
Dimethyl-pentyl-furandodecanoic acid	50.86	***	−0.99	38.49	***	0.91
Octadecatrienoyl-sn-glycerol	1.99	***	0.94	2.27	***	0.93
Aminolipid, isomer 1	1.87	***	0.88	1.87	***	0.98
Aminolipid, isomer 2	1.38	***	0.93	5.46	***	0.97
Octadecatrienoic acid, 2,3-bis(acetyloxy)propyl ester	1.59	***	0.72	1.13	n.s.	0.62
Octadecatrienoic acid, isomer 4	1.67	***	0.99	1.67	***	0.88
Octacosanedioic acid	91.93	***	1.00	200.32	***	0.99
Tricosatrienoic acid	92.91	***	0.98	128.77	***	0.99
Dioxooctacosanoic acid, isomer 1	87.02	***	0.99	131.44	***	0.99
Pentadecenyl-phenol	220.92	***	0.98	329.84	***	0.98
Dimethyloctacosanedioic acid	113.35	***	1.00	248.82	***	1.00
Phenolic lipid 1	−3.57	***	−0.98	−5.02	***	−0.99
Butenedioic acid, ditridecyl ester, isomer 1	54.63	***	1.00	62.60	***	1.00
Heptadecenyl-phenol	221.76	***	0.98	346.67	***	0.94
Dioxooctacosanoic acid, isomer 2	51.79	***	0.99	96.63	***	0.99
Phenolic lipid 2	39.70	***	0.99	90.10	***	0.99
Oxidised phosphatidylcholine	1.20	***	0.88	1.21	***	0.81
Butenedioic acid, ditridecyl ester, isomer 2	38.21	***	1.00	47.35	***	0.99
Unknown lipid 1	36.68	***	0.99	163.95	***	1.00
Unknown lipid 2, isomer 1	4.89	***	0.99	5.46	***	0.98
Unknown lipid 3, isomer 1	25.37	***	1.00	36.95	***	0.99
Unknown lipid 2, isomer 2	18.59	***	0.99	102.85	***	1.00
Unknown lipid 4, isomer 1	18.93	***	0.99	185.42	***	1.00
Unknown lipid 3, isomer 2	1.32	***	0.81	2.13	***	0.91
Unknown lipid 2, isomer 3	29.14	***	0.98	160.46	***	0.99
Unknown lipid 4, isomer 2	24.66	***	0.99	184.04	***	1.00
Sum of caffeoylquinic acids	4.05	***		3.79	***	
Sum of flavonoids	−1.04	n.s.		−1.46	***	
Sum of phenolic compounds	1.42	***		1.09	n.s.	
Sum of triterpenoid glycosides	−1.20	***		−1.31	**	
Sum of lipids	3.03	***		4.50	***	

^a^ Ratio of the relative content of metabolite: positive value—tubular flowers > ligulate flowers; negative value—tubular flowers < ligulate flowers. ^b^ Significance of differences: *—*p* < 0.05; **—*p* < 0.01; ***—*p* < 0.001; n.s.—not significant. ^c^ Correlation with orthogonal component from S-plot data of OPLS model.

**Table 6:** Differences between two *Calendula officinalis* cultivars, ‘Paradise Garden’ (PG) and ‘Golden Sea’ (GS), in the relative content of major metabolites in inflorescences, ligulate and tubular flowers. The relative content of the flower metabolites was recalculated by taking into account their proportions in the inflorescences and then combining them (Table S3). The content of metabolites in inflorescences was determined as the sum of the content in ligulate and tubular flowers. Statistical differences were detected using Student’s *t*-test.

Metabolites	Inflorescences		Tubular Flowers		Ligulate Flowers	
	GS/PG		GS/PG		GS/PG	
	Ratio ^a^	*t*-Test ^b^	Ratio	*t*-Test	Ratio	*t*-Test
	(fold)	*p*	(fold)	*p*	(fold)	*p*
3-*O*-Caffeoylquinic acid (Neochlorogenic acid)	1.68	***	1.67	***	1.69	**
5-*O*-Caffeoylquinic acid (Chlorogenic acid)	2.35	***	2.22	***	2.80	***
4-*O*-Caffeoylquinic acid (Cryptochlorogenic acid)	1.56	***	1.45	***	1.86	***
Quercetin-3-*O*-rutinosyl-rhamnoside	−1.07	n.s.	−1.05	n.s.	−1.08	n.s.
Quercetin-3-*O*-β-D-rutinoside (Rutin)	1.42	***	1.71	**	1.01	n.s.
Isorhamnetin-3-*O*-rutinosyl-rhamnoside	1.14	n.s.	1.16	n.s.	1.13	n.s.
Kaempferol-3-*O*-rutinoside	3.36	***	2.69	***	3.93	***
Quercetin-3-*O*-glucoside (Isoquercitrin)	1.41	***	1.24	**	2.20	***
Quercetin-3-*O*-rhamnosyl-glucoside	1.29	***	1.10	n.s.	1.40	***
Isorhamnetin-3-*O*-rutinoside (Narcissin)	1.49	***	1.25	**	1.59	***
Isorhamnetin 3-*O*-rhamnopyranosyl-glucopyranoside	1.35	***	1.24	**	1.67	***
3,5-Di-*O*-caffeoylquinic acid	1.24	**	1.38	**	−1.10	n.s.
Tris-trans-*p*-coumaroyl-spermine	1.44	***	1.45	**	1.21	n.s.
Isorhamnetin-malonyl-hexoside	1.36	**	1.20	**	1.54	**
Tetra-trans-*p*-coumaroyl-spermine	1.51	***	1.52	***	1.15	n.s.
Calendulaglycoside A	1.11	n.s.	1.23	**	1.06	n.s.
Calendulaglycoside B	1.11	n.s.	1.29	**	1.02	n.s.
Calenduloside G	1.26	**	1.31	**	1.21	*
Acetyloleanolic acid-glucuronide-hexoside	1.79	***	2.38	***	1.54	***
Calendulaglycoside C	−1.53	***	−1.02	n.s.	−1.79	**
Calenduloside F	−1.28	***	1.13	n.s.	−1.59	**
Calenduloside E (Oleanolic acid-glucuronide)	−1.46	***	−1.11	n.s.	−2.03	***
Trihydroxyoctadecadienoic acid	−1.28	***	1.11	n.s.	−2.10	***
Octadecatrienoic acid, isomer 1	1.75	***	1.89	**	1.63	**
Dehydrophytosphingosine	−1.86	***	1.13	n.s.	−3.41	***
Oxooctadecadienoic acid, isomer 1	1.09	n.s.	1.69	**	−1.42	***
Oxooctadecadienoic acid, isomer 2	1.50	***	1.75	***	1.31	**
Octadecatrienoic acid, isomer 2	1.66	***	2.27	***	1.13	n.s.
Dimethyl-pentyl-furandecanoic acid, isomer 1	1.54	**	1.57	**	−1.12	n.s.
Dimethyl-pentyl-furandecanoic acid, isomer 2	1.57	***	1.58	**	−1.85	**
Octadecatrienoic acid, isomer 3	1.43	**	1.77	**	−1.01	n.s.
Hydroxyoctadecatrienoyl-carnitine	2.03	***	2.08	***	−1.28	*
Dimethyl-pentyl-furandodecanoic acid	1.37	***	1.37	***	1.40	***
Octadecatrienoyl-sn-glycerol	1.22	***	1.42	***	−1.04	n.s.
Aminolipid, isomer 1	1.13	n.s.	1.26	**	−1.03	n.s.
Aminolipid, isomer 2	−1.18	**	1.49	**	−3.42	***
Octadecatrienoic acid, 2,3-bis(acetyloxy)propyl ester	−1.13	n.s.	−1.18	**	−1.09	n.s.
Octadecatrienoic acid, isomer 4	1.48	***	1.66	***	1.29	*
Octacosanedioic acid	2.15	**	2.18	**	−1.29	n.s.
Tricosatrienoic acid	2.09	***	2.11	***	1.18	n.s.
Dioxooctacosanoic acid, isomer 1	1.48	***	1.49	**	−1.31	*
Pentadecenyl-phenol	1.56	***	1.57	***	−1.23	n.s.
Dimethyloctacosanedioic acid	2.19	***	2.21	***	−1.28	*
Phenolic lipid 1	1.13	n.s.	1.05	n.s.	1.14	n.s.
Butenedioic acid, ditridecyl ester, isomer 1	1.52	***	1.53	***	1.04	n.s.
Heptadecenyl-phenol	2.38	***	2.39	***	1.18	n.s.
Dioxooctacosanoic acid, isomer 2	1.35	**	1.37	**	−1.75	**
Phenolic lipid 2	1.32	**	1.36	***	−2.16	**
Oxidised phosphatidylcholine	1.26	***	1.44	***	1.11	n.s.
Butenedioic acid, ditridecyl ester, isomer 2	1.52	***	1.55	**	−1.03	n.s.
Unknown lipid 1	1.38	**	1.43	**	−4.04	**
Unknown lipid 2, isomer 1	1.39	*	1.51	**	1.05	n.s.
Unknown lipid 3, isomer 1	1.94	***	1.99	***	1.06	n.s.
Unknown lipid 2, isomer 2	1.66	***	1.78	**	−4.00	***
Unknown lipid 4, isomer 1	1.58	***	1.71	***	−7.41	**
Unknown lipid 3, isomer 2	1.47	***	2.06	**	−1.01	n.s.
Unknown lipid 2, isomer 3	1.45	***	1.52	***	−4.68	**
Unknown lipid 4, isomer 2	1.56	***	1.65	***	−5.84	***
Sum of caffeoylquinic acids	1.50	***	1.58	***	1.31	**
Sum of flavonoids	1.32	***	1.25	***	1.37	***
Sum phenolic compounds	1.36	***	1.36	***	1.36	***
Sum of triterpenoid glycosides	1.12	n.s.	1.24	**	1.05	n.s.
Sum of lipids	1.33	***	1.59	***	−1.20	***

^a^ Ratio of the relative content of the metabolite: positive value—GS > PG; negative value—GS < PG. ^b^ Significance of differences: *—*p* < 0.05; **—*p* < 0.01; ***—*p* < 0.001; n.s.—not significant.

## Data Availability

The data are contained within the article and Supplementary Material.

## Supplementary Materials

The following supporting information can be downloaded at https://www.mdpi.com/article/10.3390/metabo14030140/s1. Figure S1: UPLC-UV profile of phenolic compounds, registered at λ 240–400 nm. Table S1: Some characteristics of the two cultivars of *Calendula officinalis*, ‘Paradise Garden’ and ‘Golden Sea’, used in this study. Table S2: Additional information to MS/MS characterisation of lipids in extract from *Calendula officinalis* flowers. Table S3: Differences in the content of the metabolites between the two cultivars of *Calendula officinalis*, ‘Paradise Garden’ and ‘Golden Sea’, for the ligulate and tubular flowers.
