# Supplementary material for: Comparative Metabolomics of Ligulate and Tubular Flowers of Two Cultivars of Calendula officinalis L
Source: Metabolites. 2024 Feb 26;14(3):140. doi: 10.3390/metabo14030140 (PMC10972464; doi:10.3390/metabo14030140)
Supplement: Supplementary file 1 [file metabolites-14-00140-s001.zip › metabolites-2876807-supplementary.pdf]

metabolites

MDPI

Supplementary Materials

Comparative Metabolomics of Ligulate and Tubular Flowers of Two Cultivars of *Calendula officinalis* L.

Vladimir Ossipov, Firdaus Khazieva, Dmitry Baleev, Juha-Pekka Salminen and Nikolay Sidelnikov

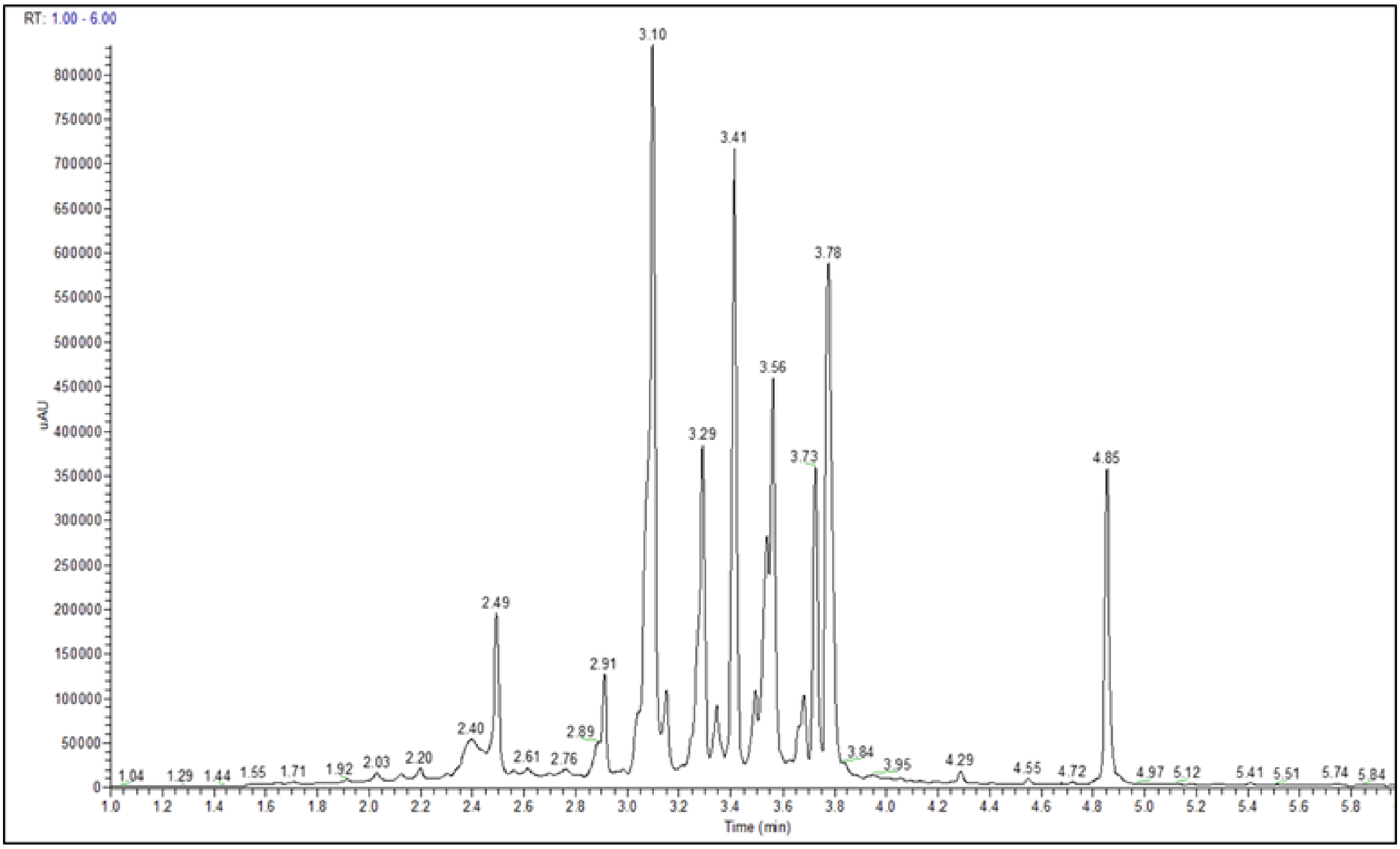

Figure S1. UPLC-UV profile of phenolic compounds, registered at λ 240-400 nm.

Table S1. Some characteristics of the two cultivars of *Calendula officinalis*, 'Paradise Garden' and 'Golden Sea', used in the study.

Characteristics of Cultivar	Paradise Garden (PG)		Golden Sea (GS)		PC vs GS
	Mean value	Standard error	Mean value	Standard error	t-test *p*
Plant height, cm	57.3	2.03	56.2	2.6	n.s.
Number of branches	6.6	0.43	6.38	0.3	n.s.
Number of leaves	28.9	1.26	31.6	1.5	*
Seed yield, kg/ha	798.3	52.0	887.6	52.6	*
Inflorescences characteristics:					
Diameter, cm	6.68	0.38	6.6	0.40	n.s.
Weight (raw), g	3.12	0.09	3.17	0.10	n.s.
Productivity, kg/ha	1853.9	163.7	2001.5	164.1	n.s.
Inflorescence parts (%):					
Receptacle	42.0	7	34.6	15	n.s.

Ligulate flowers	35.6	9	34.6	22	n.s.
Tubular flowers	22.4	9	30.8	10	**

n.s., not significant; *, $p < 0.05$; **, $p < 0.01$.

Table S2. Additional information to MS/MS fragmentation of lipids found in *Calendula officinalis* flowers.

Code	RT (min)	$[M+H]^+$ (*m/z*)	$[M+Na]^+$ (*m/z*)	MS/MS fragmentation of parent ion $[M+H]^+$: fragment (*m/z*), intensity (%), $[ion]^+$
L1	Trihydroxyoctadecadienoic acid	329.2325	351.2134	311.2210 (75) $[M-H_2O+H]^+$, 293.2106 (100) $[M-2H_2O+H]^+$, 275.2004 (9.5) $[M-3H_2O+H]^+$
L2	Octadecatrienoic acid, isomer 1	279.2319		261.2213 (40), 243.2108 (19)
L3	Dehydrophytosphingosine	316.2844		298.2736 (15) $[M-H_2O+H]^+$, 280.2826 (10) $[M-2H_2O+H]^+$, 60.0453 (100)
L4	Oxooctadecadienoic acid, isomer 1	295.2274		277.2155 (100) [Octadecadienoic acid+H]$^+$, 259.2052 (13) [Octadecapentenone+H]$^+$
L5	Oxooctadecadienoic acid, isomer 2	295.2274		277.2155 (100) [Octadecadienoic acid+H]$^+$, 259.2052 (13) [Octadecapentenone+H]$^+$
L6	Octadecatrienoic acid, isomer 2	279.2317		261.2213 (43), 243.2108 (17)
L7	Dimethyl-pentyl-furandecanoic acid, isomer 1	337.2738	359.2555	319.2632 (16) $[M-H_2O+H]^+$, 301.2518 (37) $[M-2H_2O+H]^+$, 247.2953 (9)
L8	Dimethyl-pentyl-furandecanoic acid, isomer 2	337.2738	359.2555	319.2632 (16) $[M-H_2O+H]^+$, 301.2518 (32) $[M-2H_2O+H]^+$, 247.2059 (11)
L9	Octadecatrienoic acid, isomer 3	279.2316		261.2213 (39), 243.2108 (18)
L10	Hydroxyoctadecatrienoyl-carnitine	438.3220		379.2468 (11) [Trihydroxydocosapentaenoic acid+H]$^+$
L11	Dimethyl-pentyl-furandodecanoic acid	365.3046	387.2864	347.2946 (100) $[M-H_2O+H]^+$, 329.2472 (30)
L12	Octadecatrienoyl-sn-glycerol	353.2686		335.2579 (60) $[M-H_2O+H]^+$, 261.2210 (83)
L13	Aminolipid, isomer 1	473.2395		225.1846 (26) [Tetradecadienoic acid+H]$^+$, 207.1740 (21) [Tetradecatrienal+H]$^+$, 189.1634 (29) [Diaminononanoic acid+H]$^+$, 159.0288 (10) [Hydroxy-hexadienedioic acid+H]$^+$, 145.0493 (15) [Hexenedioic acid+H]$^+$, 133.1011 (13) [Diaminopentanoic acid+H]$^+$, 85.0654 (100) [Pentenone+H]$^+$
L14	Aminolipid, isomer 2	473.2397		225.1846 (26) [Tetradecadienoic acid+H]$^+$, 207.1740 (21) [Tetradecatrienal+H]$^+$, 189.1634 (29) [Diaminononanoic acid+H]$^+$, 159.0288 (10) [Hydroxy-hexadienedioic acid+H]$^+$, 145.0493 (15) [Hexenedioic acid+H]$^+$, 133.1011 (13) [Diaminopentanoic acid+H]$^+$, 85.0654 (100) [Pentenone+H]$^+$
L15	Octadecatrienoic acid, 2,3-bis(acetyloxy)propyl ester	437.2906	461.2872	247.1692 (15) [Hexadecendiynoic acid+H]$^+$, 233.1534 (11) [Pentadecendiynoic acid+H]$^+$, 203.1793 (74), 191.1793 (54) [Trimethylundecatetraene+H]$^+$, 119.0857 (94), 95.0860 (100) [Heptatriene+H]$^+$

L16	Octadecatrienoic acid, isomer 4	279.2317		261.2213 (45), 243.2108 (22)
L17	Octacosanedioic acid	455.4093	477.3910	419.3877 (62) [Octacosatrienoic acid+H]$^+$, 365.3410 (40) [Tetracosadienoic acid+H]$^+$, 195.0860 (100), 81.0705 (90)$^+$, 67.0550 (77)
L18	Tricosatrienoic acid	349.3095	371.2919	331.2990 (21) [M-H2O+H]$^+$, 261.2209 (51) [Trimethylpentadecatetraenone+H]+, 95.0860 (67) [Heptatriene+H]$^+$, 81.0705 (90), 67.0550 (100)
L19	Dioxooctacosanoic acid, isomer 1	453.3935		435.3820 [M-H2O+H]$^+$, 349.3094 (56), 295.2628 (72) [Methyloctadecadienoic acid+H]$^+$, 113.0600 (48) [Hexadienoic acid+H]$^+$, 109.1015 (44) [Octatriene+H]$^+$, 95.0859 (92) [Heptatriene+H]$^+$, 81.0705 (100) [C6H9]$^+$
L20	Pentadecenyl-phenol	303.2680		285.2573 (62) [M-H2O+H]$^+$, 221.1898 (35) [Nonylphenol+H]$^+$, 163.1479 (73) [Hexylbenzene+H]$^+$
L21	Dimethyloctacosanedioic acid	483.4409	505.4217	465.4303 (84) [M-H2O+H]$^+$, 393.3723 (12) [Hexacosadienoic acid+H]$^+$
L22	Phenolic lipid 1	407.3153		207.1377 (95) [Phenyl-heptanoic acid+H]$^+$, 179.1064 (100) [Phenyl-pentanoic acid+H]$^+$, 161.0959 (20) [Phenyl-pentenal+H]$^+$, 133.1012 (55)
L23	Butenedioic acid, ditridecyl ester, isomer 1	481.4245	503.4062	463.4138 (71) [M-H2O+H]$^+$, 409.3671 (32) [Oxo-hexacosenoic acid+H]$^+$, 377.3409 (52) [Trimethyldocosatrienoic acid+H]$^+$, 323.2939 (50) [Methyleicosadienoic acid+H]$^+$, 309.2780 (33) [Eicosadienoic acid+H]$^+$, 113.0599 (44) [Hexadienoic acid+H]$^+$, 81.0705 (100)
L24	Heptadecenyl-phenol	331.2992		313.2887 (90) [M-H2O+H]$^+$, 239.2366 (38) [Hexadecadienol+H]$^+$, 109.1015 (22) [Octatriene+H]$^+$, 95.0880 (49) [Heptatriene+H]$^+$, 81.0705 (37), 67.0550 (33), 57.0708 (100)
L25	Dioxooctacosanoic acid, isomer 2	453.3935	475.3752	435.3821 (46) [M-H2O+H]$^+$, 349.3094 (53), 295.2630 (29), 113.0602 (44) [Hexadienoic acid+H]$^+$, 109.1014 (40) [Octatriene+H]$^+$, 95.0857 (90) [Heptatriene+H]$^+$, 81.0705 (100), 67.0550 (93)
L26	Phenolic lipid 2	473.3627		273.1848 (100) [Octadecatrienoic acid+H]$^+$, 247.1324 (59) [Hydroxypentadecadien-diynoic acid+H]$^+$, 245.1532 (62) [Hexadecatetraenynoic acid+H]$^+$, 189.1272 (88) [Tridecendiynal+H]$^+$, 179.1063 (34) [Hydroxy-phenylpentanone+H]$^+$
L27	Oxidized phosphatidylcholine	758.5676		429.3724 (100), 184.0731(54), 124.9999 (35), 86.0970 (48)
L28	Butenedioic acid, ditridecyl ester, isomer 2	481.4247	503.4066	463.4139 (77) [M-H2O+H]$^+$, 409.3671 (32) [Oxo-hexacosenoic acid+H]$^+$, 377.3409 (57) [Trimethyldocosatrienoic acid+H]$^+$, 323.2937 (67) [Methyleicosadienoic acid+H]$^+$, 95.0860 (90) [Heptatriene+H]$^+$, 81.0705 (100), 67.0550 (91)
L29	Unknown lipid 1	465.3934	487.3748	379.3198 (35), 309.2785 (100) [Eicosadienoic acid+H]$^+$, 295.2626 (100) [Methyloctadecadienoic acid+H]$^+$, 113.0599 (56) Hexadienoic acid+H]$^+$, 95.0860 (44) [Heptatriene+H]$^+$, 81.0705 (49), 71.0499 (48)
L30	Unknown lipid 2, isomer 1	479.4086	501.3907	393.3354 (20) [Hexacosadienoic acid+H]$^+$, 375.3250 (44), 323.2941 (20) [Methyleicosadienoic acid+H]$^+$, 295.2626 (100) [Methyloctadecadienoic acid+H]$^+$, 95.0859 (62) [Heptatriene+H]$^+$, 81.0705 (63), 67.0550 (54)
L31	Unknown lipid 3, isomer 1	429.3723		191.1063 (12) [Dodecapentaenoic acid+H]$^+$; 165.0908 (100) [Decadiyonic acid+H]$^+$

L32	Unknown lipid 2, isomer 2	479.4098	501.3903	393.3354 (30) [Hexacosadienoic acid+H]+, 375.3250 (44), 323.2940 (100) [Methyleicosadienoic acid+H]+, 295.2626 (100) [Methyloctadecadienoic acid+H]+, 113.0599 (60) [Hexadienoic acid+H]+, 95.0859 (42) [Heptatriene+H]+, 71.0499 (51), 67.0550 (49)
L33	Unknown lipid 4, isomer 1	493.4247	515.4068	407.3511 (47), 389.3404 (39), 379.3193 (25), 337.3093 (73) [Di-methyleicosadienoic acid+H]+, 309.2783 (100) [Eicosadienoic acid+H]+, 113.0600 (70) [Hexadienoic acid+H]+, 95.0860 (69) [Heptatriene+H]+
L34	Unknown lipid 3, isomer 2	429.3722		191.1068 (12) [Dodecapentaenoic acid+H]+, 165.0909 (100) [Decadiyonic acid+H]+
L35	Unknown lipid 2, isomer 3	479.4096	501.3913	393.3355 (47) [Hexacosadienoic acid+H]+, 375.3250 (44), 323.2942 (100) [Methyleicosadienoic acid+H]+, 295.2631 (100) [Methyloctadecadienoic acid+H]+, 113.0597 (67) [Hexadienoic acid+H]+, 95.0859 (46) [Heptatriene+H]+
L36	Unknown lipid 4, isomer 2	493.4248		407.3512 (44), 389.3404 (39), 379.3193 (25), 337.3095 (100) [Dime-thyl-eicosadienoic acid+H]+, 309.2784 (97) [Eicosadienoic acid+H]+, 295.2631 (52), 113.0601 (72) [Hexadienoic acid+H]+, 95.0860 (65) [Heptatriene+H]+

Table S3. Differences in the content of the metabolites between the two cultivars of *Calendula officinalis*, "Paradise Garden" and "Golden Sea", for the ligulate and tubular flowers.

Metabolite	Ligulate Flowers 'Golden Sea' / 'Paradise Garden'			Tubular Flowers 'Golden Sea' / 'Paradise Garden'		
	Ratio[a] (fold)	t-test[b] *p*	Correlation[c] *r*	Ratio[a] (fold)	t-test[b] *p*	Correlation[c] *r*
3-*O*-Caffeoylquinic acid (Neochlorogenic acid)	1.60	**	0.91	1.22	**	0.85
5-*O*-Caffeoylquinic acid (Chlorogenic acid)	2.65	***	0.96	1.63	**	0.87
4-*O*-Caffeoylquinic acid (Cryptochlorogenic acid)	1.76	***	0.88	1.06	n.s.	0.63
Quercetin-3-*O*-rutinosyl-rhamnoside	-1.14	n.s.	-0.69	-1.43	n.s.	-0.23
Quercetin-3-*O*-β-D-rutinoside (Rutin)	-1.05	n.s.	-0.81	1.25	*	-0.87
Isorhamnetin-3-*O*-rutinosyl-rhamnoside	1.07	n.s.	0.46	-1.17	n.s.	-0.86
Kaempferol-3-*O*-rutinoside	3.72	***	0.80	1.97	***	0.85
Quercetin-3-*O*-glucoside (Isoquercitrin)	2.08	***	0.89	-1.10	n.s.	-0.66
Quercetin-3-*O*-rhamnosyl-glucoside	1.32	***	0.95	-1.24	**	-0.91
Isorhamnetin-3-*O*-rutinoside (Narcissin)	1.51	***	0.65	−1.09	*	−0.84
Isorhamnetin 3-*O*-rhamnopyranosyl-gluco-pyranoside	1.58	**	0.82	−1.10	n.s.	−0.89
3,5-Di-*O*-caffeoylquinic acid	−1.17	*	−0.33	1.02	n.s.	−0.20
Tris-trans-p-coumaroyl-spermine	1.14	n.s.	0.13	1.06	n.s.	0.63
Isorhamnetin-malonyl-hexoside	1.46	**	0.85	−1.14	**	−0.96
Tetra-trans-p-coumaroyl-spermine	1.08	n.s.	0.17	1.11	n.s.	0.73
Calendulaglycoside A	1.01	n.s.	0.11	−1.11	n.s.	−0.44
Calendulaglycoside B	−1.03	n.s.	−0.45	−1.05	n.s.	−0.41
Calenduloside G	1.15	n.s.	0.50	−1.04	n.s.	0.41
Acetyloleanolic acid-glucuronide-hexoside	1.45	***	0.84	1.76	***	0.85
Calendulaglycoside C	−1.89	***	−0.96	−1.38	**	−0.85
Calenduloside F	−1.68	**	−0.96	−1.21	n.s.	−0.80

Calenduloside E (Oleanolic acid-glucuronide)	−2.15	***	−0.90	−1.52	**	−0.83
Trihydroxyoctadecadienoic acid	−2.22	***	−0.99	−1.23	**	−0.96
Octadecatrienoic acid, isomer 1	1.54	**	0.86	1.38	**	0.92
Dehydrophytosphingosine	−3.60	***	−0.99	−1.21	*	−0.87
Oxooctadecadienoic acid, isomer 1	−1.50	***	−0.99	1.24	*	0.86
Oxooctadecadienoic acid, isomer 2	1.24	n.s.	0.78	1.29	**	0.89
Octadecatrienoic acid, isomer 2	1.07	n.s.	−0.58	1.66	**	0.94
Dimethyl-pentyl-furandecanoic acid, isomer 1	−1.18	n.s.	−0.61	1.15	n.s.	0.05
Dimethyl-pentyl-furandecanoic acid, isomer 2	−1.95	***	−0.82	1.16	n.s.	0.54
Octadecatrienoic acid, isomer 3	−1.06	n.s.	0.49	1.30	n.s.	0.71
Hydroxyoctadecatrienoyl-carnitine	−1.36	*	−0.87	1.53	***	0.90
Dimethyl-pentyl-furandodecanoic acid	1.32	**	0.98	1.00	n.s.	0.48
Octadecatrienoyl-sn-glycerol	−1.10	n.s.	0.51	1.04	n.s.	0.55
Aminolipid, isomer 1	−1.09	n.s.	−0.66	−1.08	n.s.	−0.11
Aminolipid, isomer 2	−3.62	***	−0.82	1.09	n.s.	0.93
Octadecatrienoic acid, 2,3-bis(acetyloxy)propyl ester	−1.15	n.s.	−0.61	−1.62	***	−0.88
Octadecatrienoic acid, isomer 4	1.22	n.s.	0.62	1.22	***	0.84
Octacosanedioic acid	−1.37	*	−0.86	1.60	**	0.93
Tricosatrienoic acid	1.11	n.s.	0.25	1.54	***	0.82
Dioxooctacosanoic acid, isomer 1	−1.38	*	−0.82	1.09	n.s.	0.35
Pentadecenyl-phenol	−1.30	n.s.	−0.48	1.15	n.s.	0.29
Dimethyloctacosanedioic acid	−1.35	*	−0.81	1.62	***	0.95
Phenolic lipid 1	1.08	n.s.	0.07	−1.30	n.s.	−0.24
Butenedioic acid, ditridecyl ester, isomer 1	−1.02	n.s.	−0.26	1.12	n.s.	0.79
Heptadecenyl-phenol	1.12	n.s.	0.32	1.75	***	0.85
Dioxooctacosanoic acid, isomer 2	−1.85	**	−0.85	1.01	n.s.	0.10
Phenolic lipid 2	−2.28	**	−0.88	−1.00	n.s.	−0.08
Oxidized phosphatidylcholine	1.05	n.s.	0.55	1.06	n.s.	0.06
Butenedioic acid, ditridecyl ester, isomer 2	−1.09	n.s.	−0.71	1.13	n.s.	0.71
Unknown lipid 1	−4.28	**	−0.85	1.05	n.s.	0.10
Unknown lipid 2, isomer 1	−1.01	n.s.	−0.36	1.10	n.s.	0.35
Unknown lipid 3, isomer 1	1.00	n.s.	0.27	1.46	**	0.86
Unknown lipid 2, isomer 2	−4.23	***	−0.81	1.31	*	0.81
Unknown lipid 4, isomer 1	−7.83	**	−0.83	1.25	**	0.86
Unknown lipid 3, isomer 2	−1.07	n.s.	−0.36	1.51	***	0.95
Unknown lipid 2, isomer 3	−4.95	**	−0.80	1.11	n.s.	0.41
Unknown lipid 4, isomer 2	−6.18	***	−0.83	1.21	**	0.83
Sum of caffeoylquinic acids	1.24	**		1.16	*	
Sum of flavonoids	1.29	***		−1.09	**	
Sum of phenolic compounds	1.29	***		−1.00	n.s.	
Sum of triterpenoid glycosides	−1.01	n.s.		−1.10	n.s.	
Sum of lipids	−1.27	***		1.17	**	

[a]Ratio of the relative content of the metabolite, positive value – GS > PG, negative value – GS < PG.

[b]Significance of differences: * - $p < 0.05$, ** - $p < 0.01$, *** - $p < 0.001$; n.s., not significant

[c]Correlation with orthogonal component from S-plot data of OPLS model
